# The Prognostic Impact of Enteropathy and Liver Disease in Common Variable Immunodeficiency: A Retrospective Cohort Study

**DOI:** 10.14309/ctg.0000000000000952

**Published:** 2025-12-01

**Authors:** José Miranda-Bautista, Helena Martínez-Lozano, Marisa Di Natale, María Alejandra Mejía González, Ignacio Marín-Jiménez, Paloma Sánchez-Mateos, María Isabel Peligros Gómez, Diego Rincón, Rafael Bañares, Luis Menchén

**Affiliations:** 1 Department of Gastroenterology and Hepatology, Hospital General Universitario Gregorio Marañón, Madrid, Spain;; 2 Instituto de Investigación Sanitaria Gregorio Marañón (IiSGM), Madrid, Spain;; 3 Immunology Service, Hospital General Universitario Gregorio Marañón, Madrid, Spain;; 4 Pathology Service, Hospital General Universitario Gregorio Marañón, Madrid, Spain;; 5 Departamento de Medicina, Universidad Complutense de Madrid, Madrid, Spain;; 6 Centro de Investigación en Red de Enfermedades Hepáticas y Digestivas, Madrid, Spain.

## Abstract

**INTRODUCTION:**

Up to one-third of the patients diagnosed with common variable immunodeficiency (CVID) may develop gastrointestinal (GI) and hepatic manifestations. This study aimed to evaluate the prognostic significance of enteropathy and liver disease in patients with CVID.

**METHODS:**

We conducted a retrospective study including all consecutive adult patients with CVID followed in a tertiary care center in Spain from January 1990 to January 2023. A diagnosis of CVID-associated enteropathy (CVID-E) and CVID-associated liver involvement (CVID-L) was established when objective clinical, endoscopic, histologic, radiologic or hemodynamic findings were present. Relevant prognostic outcomes and their risk factors were studied, including survival, GI infections, and GI cancer.

**RESULTS:**

Eighty-nine patients with confirmed CVID were included, 26 of them (29.2%) had CVID-E and 23 (25.8%) had CVID-L. Nineteen (73.1%) patients with CVID-E suffered from GI infections, while 12 (46.2%) presented concurrent liver involvement. In comparison with the rest of the cohort, patients with CVID-E had more frequently liver involvement, GI infections, and GI cancer. Multivariate analysis identified CVID-E as an independent risk factor for GI infections. Twelve (52.2%) patients with CVID-L concurrently exhibited CVID-E, and patients with CVID-L presented more CVID-E, splenomegaly, and a trend toward more GI cancer and GI infections. CVID-L and age at CVID diagnosis emerged as independent risk factors for mortality.

**DISCUSSION:**

GI and hepatic involvements are common in patients with CVID and frequently occur together. These manifestations significantly affect the disease course, increasing the risk of GI infections, GI malignancy, and, in the case of liver disease, mortality.

## Graphical abstract

**Figure:**
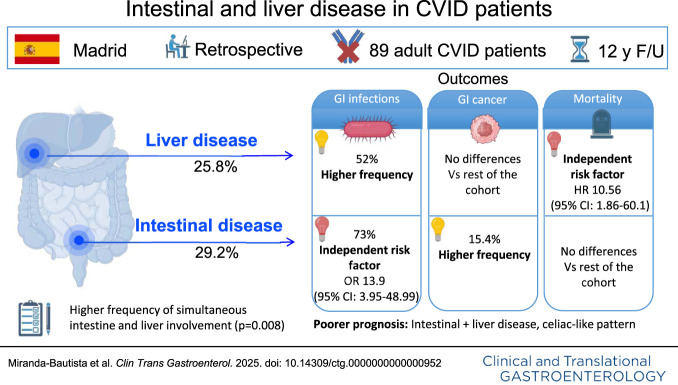

## INTRODUCTION

Common variable immunodeficiency (CVID) is the most common symptomatic primary immunodeficiency disorder in adults (1,2) and is characterized by a decrease in immunoglobulin (Ig) G, Ig A, and/or Ig M production. B-cell dysfunction plays a significant role in its pathogenesis (3–6); however, other mechanisms, such as T-cell deficiency, monocyte or macrophage hyperactivity, and systemic inflammation are also relevant as pathogenic contributing factors. Thus, the pathophysiology of the disease is considered complex and multifactorial, and genetic, epigenetic, and environmental factors may influence its development. Not surprisingly, the clinical manifestations of CVID are highly heterogeneous. In fact, although most patients experience infectious complications, up to 70% of patients also have autoimmune or inflammatory disorders (7). Accordingly, a recent consensus conference has established 4 CVID clinical phenotypes (8): no other disease-related complications (previously “infectious only”), cytopenias, polyclonal lymphoproliferation, and unexplained persistent enteropathy (9).

As shown in the consensus, the intestine is, in fact, an organ of relatively frequent involvement. CVID-associated enteropathy (CVID-E), which occurs in 9%–20% of patients with CVID (10), consists in an inflammatory disorder of the intestinal mucosa and wall that may affect any segment of the gastrointestinal (GI) tract. Although increasing efforts have been made to unravel its pathogenesis, no definite hypothesis has been confirmed. Low Ig A levels that trigger GI infections as *Salmonella*, *Campylobacter*, or *Giardia* is believed to be crucial, together with norovirus (11) or *Acinetobacter baumanii* infection (12). The mononuclear infiltrate observed in the lamina *propria* may trigger mucosal injury, being also responsible of the imbalance in T-cell homeostasis. Mucosal inflammation is observed during endoscopic procedures or in the biopsies, as along with such as villous atrophy, intraepithelial lymphocytosis, or granulomas in the small intestine and cryptic abscesses in the colon (7). Thus, CVID enteropathy may mimic celiac disease and inflammatory bowel disease (IBD), making challenging their differentiation. In fact, differentiation of celiac disease and CVID is of great importance and challenging. Thirty-one to 87% of patients with CVID may have an increased number of intraepithelial lymphocytes or villous atrophy (13–16), not discriminated by HLA-DQ2 or DQ-8 testing (17), and celiac antibodies may not be reliable because of the intrinsic impairment of Ig synthesis in these patients. Finally, a gluten-free diet can show partial (18,19) or residual efficacy (13,20).

CVID-associated liver disease (CVID-L) is observed in 11%–42% of patients with CVID (21–23). The spectrum of liver involvement in these patients ranges from mildly elevated liver enzymes to portal hypertension, hepatic decompensation, and liver failure (24). The most frequently observed histological manifestation is nodular regenerative hyperplasia (NRH), especially in patients with portal vein thrombosis or receiving thiopurines (25). Noncaseating granulomas or hepatitis may also be seen. The pathogenesis of these findings is unknown, but it is hypothesized that an increased intestinal permeability may develop bacterial translocation, triggering liver inflammation through toll-like receptor activation (17,26). CVID-L is recognized as an associated disease to porto-sinusoidal vascular disease (PSVD). Interestingly, up to 10% of patients with PSVD have some form of immune system disorder (27).

Patients with CVID are at higher risk of death than age-matched and sex-matched controls (7). Although infections are frequent complications in these patients, noninfectious phenotype also importantly increases mortality (7), in part because of the lack of efficacy of parenteral Ig administration in this scenario (21). Although intestinal and hepatic disease in the context of CVID is believed to worsen prognosis (7,23,25), there is not appropriate real-life information from longitudinally followed series of CVID. Specifically, GI complications are of great concern because of its high incidence in this setting. Although GI infections are the most common GI complication in patients with CVID, they are rarely described in the CVID-E and CVID-L context; GI cancer is especially frequent and associated with poor prognosis (28). Moreover, the prognostic influence of liver involvement is also poorly described.

Therefore, intestinal and liver disease in patients with CVID may influence patients' prognosis; however, current real-life data from large longitudinal series are scarce. Thus, we aim to describe the incidence and the clinical and prognostic implication of CVID-E and CVID-L in a large real-life series from a CVID reference center.

## METHODS

### Patients

All consecutive patients with CVID, diagnosed following European Society of Immune Deficiencies criteria (29) and followed in the Hospital General Universitario Gregorio Marañón (Madrid, Spain) from January 1990 to January 2023 were included. The Immunology Service of our institution is a referral center for the management of primary immunodeficiencies. Data on demographic variables, date of CVID diagnosis, phenotype at diagnosis, and date and cause of death were collected after revision of clinical charts. Intestinal and liver diagnostic tests, the date of CVID-E and CVID-L diagnosis, and its clinical manifestations were registered. Laboratory tests, GI infection episodes, *Helicobacter pylori* infection, GI cancer diagnosis, lymphoma of any site, surgeries, and viral hepatitis status were specifically addressed. All the performed endoscopic procedures and biopsies were reviewed. Finally, splanchnic imaging data, liver stiffness, endoscopic signs of portal hypertension, liver hemodynamics (hepatic venous pressure gradient [HVPG]), and liver histology were obtained and recorded.

All patients were followed up according to the same protocol including scheduled blood test and clinical interview every 6 months. Patients presenting with signs or symptoms of intestinal or liver disease were referred to Gastroenterology and Hepatology Department for evaluation. Thus, asymptomatic patients may have not been evaluated. Because no standardized management has been recommended for CVID-E or CVID-L, the treatment and diagnostic procedures used were performed depending on the physician's opinion and expertise. In general, patients with CVID-E have been managed according to the phenotypic pattern, following IBD guidelines; for CVID-L, management was established also following the guidelines.

### Definitions

Diagnosis of CVID: CVID was considered for inclusion with the following criteria: (i) older than 18 years; (ii) Ig G levels <5 g/L; (iii) at least 1 clinical manifestation of immune failure and at least 3 laboratory criteria related to immune deficiency; (iv) exclusion of other causes of immunodeficiency (29). All patients were receiving intravenous or subcutaneous Igs and maintained an Ig G trough level >1,000 mg/dL.

Diagnosis of CVID-E: CVID-E was established in patients presenting clinical, laboratory, or radiological signs or symptoms suspicious of GI disease, when (i) patchy intestinal inflammation (Crohn's disease-like pattern) or colonic continuous inflammation (ulcerative colitis-like pattern) was demonstrated in ileocolonoscopy, or (ii) villous atrophy was observed in duodenal biopsies (celiac-like pattern), or (iii) chronic inflammatory infiltrate was observed in colonic biopsies with normal endoscopic appearance (microscopic colitis-like pattern). Patients with GI symptoms without any evidence of intestinal inflammation or villous atrophy or patients with isolated GI infections were not considered CVID-E.

Diagnosis of CVID-L: CVID-L was established when (i) signs of portal hypertension were observed in imaging tests, hepatic vein catheterization, endoscopic procedures, or transient elastography; (ii) characteristic alterations were present in liver biopsy. These procedures were performed based on clinical judgement. Patients with isolated abnormal liver function tests or splenomegaly without evidence of structural damage or portal hypertension were not considered as CVID-L.

### Statistical analysis

Data are expressed as mean and standard deviation for quantitative variables and as absolute values and percentages for qualitative. Comparison of means was performed using a two-tailed *t* test or a Mann-Whitney U test according to data distribution. Similarly, one-way analysis of variance with a Tukey test for multiple post-test comparisons or a Kruskal–Wallis test were used as appropriate. χ^2^ was used for proportion comparisons. Time to event data were represented with Kaplan-Meier curves compared using the log-rank test. Multivariate logistic regression was used to identify independent contributors to the development of GI infections and cancer. The prognostic influence of different variables was analyzed with multivariate Cox regression. Variables included in the models were selected based on their clinical relevance and/or their statistical association in univariate analysis. Frequency of diagnosis of CVID-E and CVID-L over time was calculated as number of new diagnosis divided by the number of studied patients. Calculations were performed with IBM SPSS Statistics for Windows, version 21.0 (Armonk, NY: IBM Corp), or R version 4.3.3 (R Foundation for Statistical Computing, Vienna, Austria). A *P* value <0.05 was considered as statistically significant.

## RESULTS

### Characteristics of the whole cohort

Eighty-nine consecutive patients with confirmed CVID were included in the study. The median follow-up was 12.2 (9.7) years. Patients' characteristics are summarized in Table 1. The mean age at inclusion in the study was 50 years. The most common CVID phenotype at diagnosis was infectious (66%), followed by polyclonal lymphocytic proliferation (12.5%), cytopenia (11.3%), and enteropathy (10%).

**Table 1.** Characteristics of patients diagnosed with CVID

Variable	Overall cohort of patients with CVID (n = 89)	Patients with CVID-enteropathy (n = 26)	*P* value (enteropathy vs rest of the cohort)	Patients with CVID-liver (n = 23)	*P* value (liver vs rest of the cohort)
Male sex, n (%)	37 (41.6)	13 (50)	0.35	14 (60.9)	0.048
Age at CVID diagnosis (yr, mean (SD))	37.1 (16)	36.8 (21.1)	0.92	35 (17.6)	0.48
Splenomegaly, n (%)	37 (41.6)	12 (46.2)	0.21	19 (90.5)	0.003
GI infections (except HP), n (%)	31 (34.8)	19 (73.1)	0.0001	12 (52.2)	0.07
GI cancer (%)	5.6%	15.4%	0.02	13%	0.1
Gastric cancer (n)	3	2		1	
Colorectal cancer (n)	1	1		1	
Intestinal lymphoma (n)	1	1		1	

CVID, common variable immunodeficiency; GI, gastrointestinal; HP, *Helicobacter pylori*.

Overall, CVID-E or CVID-L were present in 37 (41.6%) cases [CVID-E: 26 (29.2%); CVID-L: 23 (25.8%)]. In 12 cases (13.5%) CVID-E and CVID-L were simultaneously diagnosed. Serological markers of past hepatitis B infection was seen in 24.7% of the patients. Five patients had received splenectomy because of refractory thrombocytopenia.

### CVID-enteropathy

The presence of CVID-E was studied in 52 patients because of suspicious signs or symptoms (58.4% of the complete cohort), and finally, 26 of them were diagnosed with CVID-E (50% of the studied patients, 29.2% of the whole cohort) (Table 1). The diagnostic rate over the time of CVID-E diagnosis was 2.94 cases per 100 patient-year (95% CI 1.82–4.49). The diagnosis of CVID-E was obtained at a median of 6.4 (15.5) years after CVID diagnosis. Mean age at CVID-E diagnosis was 42.4 (17) years. Enteropathy phenotype at diagnosis was observed in 36.4% of patients with CVID-E, and 10 out of the 26 patients with CVID-E (38.5%) were diagnosed with enteropathy before or at the same time of CVID diagnosis. There were no differences regarding age, sex, age at CVID diagnosis, and splenomegaly between patients with CVID-E and the rest of the cohort. Moreover, the proportion of patients with CVID-E who had CVID-L was greater than the observed in the rest of the cohort (46.2% vs 25.8%, *P* = 0.008).

**Figure 1.**
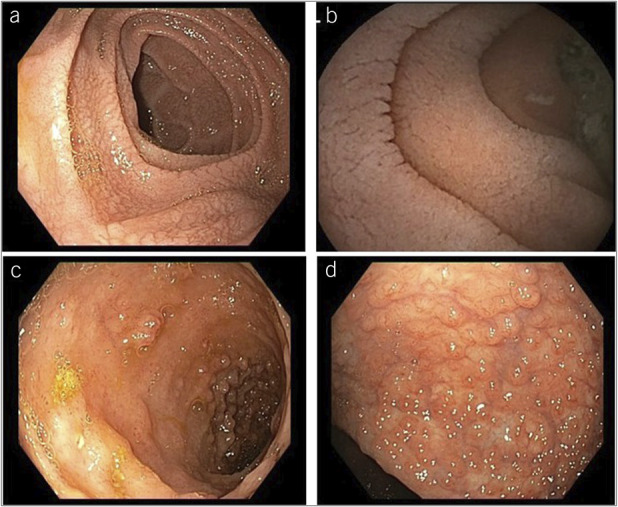
Endoscopic examples of enteropathy of common variable immunodeficiency. (**a**) Duodenal and (**b**) jejunal atrophic mucosa; (**c**) ileal inflammation with aphtous ulcers and erosions; (**d**) colonic erosions and pseudopolyps.

**Figure 2.**
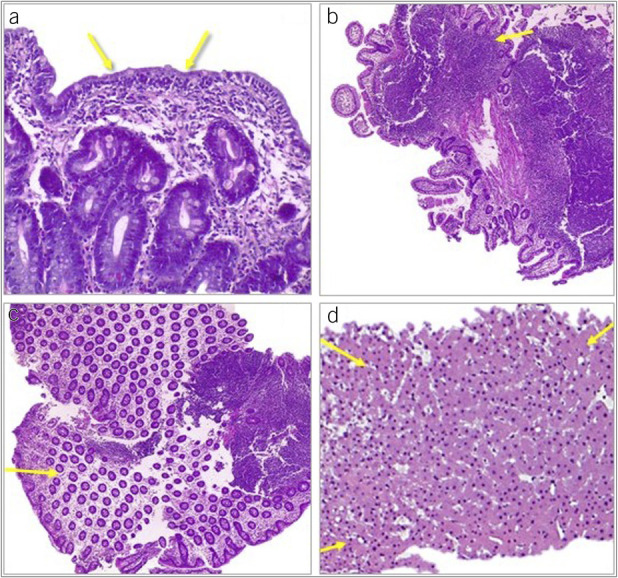
Histologic hematoxylin-eosin examples of intestinal and liver pathology of common variable immunodeficiency. (**a**) Duodenal sample showing villous atrophy and intraepithelial lymphocytosis; (**b**) ileal and (**c**) colonic chronic inflammation with lymphoid hyperplasia; (**d**) liver specimen showing nodular regenerative hyperplasia surrounded by dilated sinusoids.

Endoscopic and histologic characteristics were as follows: The most frequent observed phenotypic pattern was Crohn's disease-like [n = 12 (46.2%)], followed by ulcerative colitis-like [n = 6 (23.1%)], microscopic colitis-like [n = 4 (15.4%)], and celiac disease-like [n = 4 (15.4%)]. Duodenal villous atrophy was present in all patients with a celiac disease-like pattern as well as in a large proportion of cases with the other phenotypes (Crohn's disease-like: n = 5; ulcerative colitis-like: n = 4; and microscopic colitis-like: n = 2). Figures 1 and 2 show representative endoscopic and histologic features of patients with CVID-E, respectively.

Regarding the clinical management of patients with CVID-E, 8 patients were treated with corticosteroids, 2 with azathioprine, and 1 patient initially received infliximab, then followed by ustekinumab. In addition, 3 patients required surgical intervention. In 1 case with refractory ulcerative colitis-like pattern, a subtotal colectomy was performed; in 2 cases with Crohn's disease-like pattern ileal resection due to perforation at disease onset and terminal colostomy due to severe perianal disease were also performed. Furthermore, a patient with Crohn's disease-like pattern developed intestinal failure requiring home parenteral nutrition.

### Patients with CVID-liver disease characteristics and management

The presence of CVID-L was evaluated in 56 patients because of suspicious signs or symptoms (62.9% of the total cohort), and finally, 23 of them (41.1% of the studied patients, 25.8% of the whole cohort) were diagnosed with CVID-L at a median of 13 (10.8) years after CVID diagnosis (Table 1). Mean age at CVID-L diagnosis was 45.8 years (15). The diagnostic rate over the time of CVID-L diagnosis was 2.2 cases per 100 patient-year (95% CI 1.23–3.63). Male sex was more frequent in CVID-L patients compared with the rest of the cohort (60.9% vs 41.6%, *P* = 0.048). Spleen enlargement (90.5%, vs 50%, *P* = 0.003) and serological markers of past hepatitis B infection (52.2% vs 15.2%, *P* = 0.001) were more frequent in CVID-L. The proportion of CVID-L who also had CVID-E was also greater (52.2% vs 28.1%, *P* = 0.008). No differences were observed in the CVID phenotype at diagnosis between patients with CVID-L and the rest of the cohort.

The clinical manifestations of CVID-L were associated to portal hypertension and its complications: 9 (39.1%) patients had esophageal varices, with portal hypertension-related bleeding occurring in 3 of them. Four patients (17.4%) had ascites, 3 (13%) developed hepatic encephalopathy, 3 (13%) had hepatopulmonary syndrome, and 2 (8.7%) experienced spontaneous bacterial peritonitis.

Complementary studies were as follows: 7 patients received hepatic vein catheterization showing mild increase in HVPG [10.8 (4.8) mm Hg]. Transient liver elastography was obtained in 10 patients [mean liver stiffness 8.1 (2.7) kPa]. Histological liver assessment was available in 14 patients (60.9%). Overall, liver architecture was preserved in most cases; only 2 patients showed significant fibrosis. Three patients, one of them receiving azathioprine, presented NRH. In 2 cases, minimal portal or lobular inflammation was observed; finally, 2 patients presented with minimal histologic changes, and 2 patients had normal histologic findings. Figure 2 shows a histologic example of CVID-L.

All liver-related complications were managed according to clinical guidelines. In addition to medical and endoscopic therapy 3 patients required transjugular intrahepatic portosystemic shunt placement because of refractory portal hypertension complications (2 acute variceal bleeding and 1 portal vein thrombosis), 2 of which are still alive. No liver transplantation was performed in patients with CVID-L in our cohort. Splenectomy was performed in 4 patients to treat persistent thrombocytopenia, a procedure done more frequently than in the rest of the cohort (17.4% vs 5.6%, *P* = 0.015).

### Influence of enteric and/or liver manifestations in the natural history of CVID

GI infections, excluding *Helicobacter pylori* infection, were significantly more frequent in CVID-E (73.1% vs 34.8%, *P* = 0.0001), duodenal atrophy pattern patients (93.3% vs 45.5% in other patterns, *P* = 0.01), and also in CVID-L (52.2% vs 34.8%, *P* = 0.07). The most frequently involved agents were *Giardia lamblia*, *Campylobacter jejuni*, and *Clostridioides difficile*. Age at CVID diagnosis [OR: 0.96 (CI 95%: 0.92–0.99)] and the presence of CVID-E [OR: 13.9 (CI 95%: 3.95–48.99)] were independently associated with an increased risk of GI infections.

Four of the 5 observed GI cancers were diagnosed in the CVID-E cohort. Indeed, the incidence of GI cancer development was higher in this group of patients (15.4% vs 4.5%, *P* = 0.02).

On the contrary, lymphoma was more frequently diagnosed in CVID-L (21.7% vs 5.6% in the rest of the cohort, *P* = 0.001), but not in patients with CVID-E (*P* = 0.63).

The patients who suffered from CVID-E and CVID-L simultaneously (n = 12) suffered from more GI infections (75% vs 28.6%, *P* = 0.003) and more GI cancer than the rest of the cohort (25% vs 2.6%, *P* = 0.016).

### Mortality

**Figure 3.**
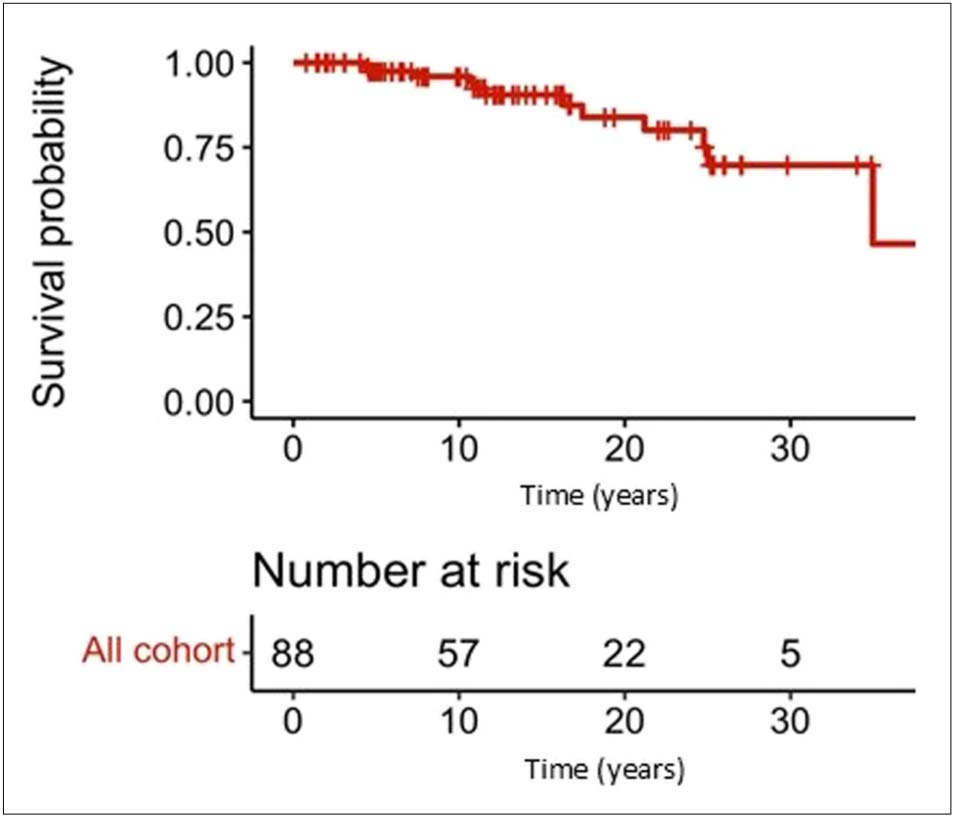
Overall survival of the whole cohort of common variable immunodeficiency patients.

At the time of the analysis (median follow-up of 12.2 (9.7) years), 12 patients (13.5%) of the patients have died, at a mean age of 62.6 (17) years. Dead patients had more likely CVID-L (58.3% vs 20.8%, *P* = 0.01) and both CVID-E and CVID-L (33.3% vs 10.4%, *P* = 0.05). The causes of death were infections (3 patients), gastric cancer (2), bladder cancer (1), liver complications (1), end-stage enteropathy (1), sudden death (1), postoperative complications (1), and unknown cause (2). Figure 3 illustrates the survival curve for the entire cohort of patients with CVID. The probability of survival was 97.5%, 95.9%, and 83.9%, at 5, 10, and 20 years from CVID diagnosis.

**Figure 4.**
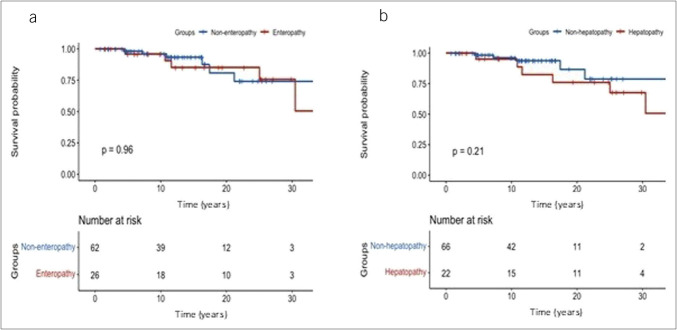
Survival probability of common variable immunodeficiency patients with (**a**) enteropathy and (**b**) liver involvement versus the rest of the cohort.

Regarding CVID-E cases, 5 patients died (19.2% vs 11.1% in the rest of the cohort, *P* = 0.32) at a mean age of 56 (23) years. The duodenal atrophy phenotype group had a higher mortality rate than the other CVID-E phenotypes (33.3% vs 0%, *P* = 0.05). Figure 4a shows the survival curve for patients with CVID-E compared with the rest of patients with CVID. No statistical differences in survival were observed comparing CVID-E and the rest of the cohort using the log-rank test.

Concerning CVID-L, 7 patients died (30.4% vs 13.5% in the rest of the cohort, *P* = 0.01), at a mean age of 51 (13) years. Survival analysis did not find differences between CVID-L and the rest of the cohort (Figure 4b).

We performed a Cox regression analysis to unravel risk factors for mortality, and we found that the presence of CVID-associated liver disease was independently associated to the risk of death in patients with CVID, together with the age at CVID diagnosis (Table 2).

**Table 2.** Risk factors of mortality in patients with CVID

Variable	Univariant HR (CI: 95%)	*P* value	Variable	Multivariant HR (CI: 95%)	*P* value
Age at CVID diagnosis	1.06 (1.03–1.1)	0.0001	Age at diagnosis	1.118 (1.06–1.19)	0.0001
Sex	1.3 (0.4–4.3)	0.662			
Enteropathy	0.99 (0.3–3.24)	0.99			
GI infections	0.71 (0.22–2.29)	0.57			
Liver involvement	2.06 (0.62–6.83)	0.24	Liver involvement	10.56 (1.86–60.1)	0.008

CVID, common variable immunodeficiency; GI, gastrointestinal.

## DISCUSSION

Although CVID was characterized several decades ago, its complete diagnosis and clinical management remain challenging. This is particularly relevant when considering the involvement of the digestive system, a relatively frequent occurrence with potentially significant clinical consequences. Here, we present a careful evaluation of these clinical patterns in one of the largest published series of CVID (13,14,20,30–34). As described above, more than a quarter of patients with CVID may develop intestinal or liver disease, and we have demonstrated that these manifestations represent also risk factors for GI infections and mortality. Of note, the patients suffering from both CVID-E and CVID-L may be a subgroup of worse prognosis.

Overall, we show that GI or liver involvement frequently appears in patients with CVID. One notable finding is that, even considering the strict diagnostic criteria used in the study—which excluded from CVID-E diagnosis those patients with GI symptoms without endoscopic or histologic signs of inflammation or with solely GI infections—the prevalence of CVID-E was greater than previously reported (10). It should be noted that GI infections were more frequent in CVID-E, indicating that the existence of an infection does not preclude a CVID-E diagnosis.

The observed intestinal involvement in CVID is heterogeneous, showing various patterns. The most frequently observed was a Crohn's disease-like pattern, although duodenal atrophy (isolated or combined with other patterns) was also frequently observed. Interestingly, duodenal atrophy was associated with more GI infections and greater mortality compared with other patterns. It must be emphasized that differentiating between duodenal CVID-E and true celiac disease is complex, and some histological findings have been suggested to better identify the former: milder intraepithelial lymphocytosis, less severity of villous atrophy (35,36), paucity of intestinal plasma cells (30), presence of follicular lymphoid hyperplasia, graft-versus-host-like crypt apoptosis, and neutrophil infiltration (13). Moreover, up to two-thirds of patients with CVID may present unspecific GI symptoms or GI infections (14,31,32,34) that may mimic the clinical manifestations of enteric involvement. Therefore, histologic evaluation is crucial, considering that macroscopic signs of inflammation are only seen in 26% of patients with CVID with GI symptoms, whereas histological abnormalities were found in 80% (13). Thus, the indication of endoscopic procedures, with special attention to obtaining biopsies, should be strongly recommended in the evaluation of these patients. It should also be noted that endoscopic or histologic GI findings may represent the first clinical observation of an otherwise unsuspected CVID. A previous pathology series showed that up to 25% of patients with CVID had a previous diagnosis of celiac disease and up to 35% had been diagnosed with IBD (30). Confirming those data, 10 patients in our CVID-E cohort (38.5%) were diagnosed with enteropathy before or at the same time as the CVID diagnosis. Physicians should be aware of this fact, and an extensive interview with the patient concerning previous infections or family history, as well as Ig measurements, should be performed at IBD or celiac disease diagnosis. Contrary to what is observed for the prevention of infections, the response of CVID-E to Ig supplementation is poor, similar to other autoimmune complications of CVID (13,37,38). It has been suggested that systemic or locally active corticosteroids may be used in the acute phase of the enteropathy because most patients may improve their symptoms or histology (13,33). Immunosuppressive drugs or biologics have been previously used, e.g., infliximab (39–42), azathioprine plus adalimumab (43,44), and the IL-12/23 blocker ustekinumab (45). In our series, only 8 patients received specific therapy, mainly corticosteroids, suggesting that the complexity of managing CVID-E patients might induce reluctance to initiate a more intensive therapeutic approach.

The second relevant finding from our study is the frequent presence of liver involvement in patients with CVID, which confers a worse prognosis. It should be noted that to obtain an accurate diagnosis of this entity, an extensive workup is necessary and should include not only possible common liver diseases (i.e., viral hepatitis, autoimmune diseases) but also have to consider PSVD, an entity characterized by the presence of unequivocal manifestations of portal hypertension and absence of cirrhosis in liver biopsy. The pathogenesis of PSVD is not well known, but in some cases, it is associated with a systemic disease, with CVID being a well-recognized cause. Importantly, liver stiffness and HVPG are clearly lower than those observed in cirrhosis (46), suggesting lesser extension of fibrosis and a presinusoidal component of portal pressure. There are some specific histological findings, especially including NRH, which may be present even in absence of abnormal liver tests (24,25,47) or symptoms. Finally, PSVD natural history includes a plethora of portal hypertension complications that may arise independently of the specific histologic features or associated disease (48). Thus, symptoms as well as signs of portal hypertension may also be seen in patients with CVID. Our data support these findings; one-third of cases had esophageal varices, some of them with variceal bleeding requiring salvage transjugular intrahepatic portosystemic shunt in 3 cases, and 41% of cases had large splenomegaly, with no case of cirrhosis in liver biopsies. One relevant finding of our study is that liver involvement, together with the age at CVID diagnosis, is an independent predictor of mortality, a fact that was previously identified by other groups (7,23,25), indicating that it should be specifically addressed. Considering the high prevalence of portal hypertension in our series and its impact on prognosis, we suggest that liver evaluation in CVID should include hemodynamics and liver biopsy.

Interestingly, 12 patients suffered from both CVID-E and CVID-L, a finding previously recognized (24). The gut-liver axis may represent one of the pathogenic explanations of this cross-relation (49). Patients with CVID present decreased alpha and beta diversity in the gut microbiome and decreased symbiotic beneficial bacteria, while an increase in pathobionts (50). Furthermore, intestinal infections and low IgA production (12) may lead to increased intestinal permeability that allows bacteria and their products to pass into the portal circulation. This circumstance may induce liver damage through several inflammatory pathways, including TLR4 activation in innate immunity cells (50,51). Thus, special attention must be paid to those patients who suffer from both intestinal and liver involvement in the context of CVID, as a higher percentage of GI infections (up to 75%), GI cancer (up to 25%), and mortality (up to 33% were dead) has been observed in our cohort. These high rates warrant stricter microbiologic and endoscopic vigilance together with close clinical and nutritional follow-up. This poor prognosis has never been addressed in the literature, to the best of our knowledge, and larger and collaborative studies are needed to better recognize the outcomes of this subgroup of patients. Igs are not useful in the management of the CVID-L scenario nor are corticosteroids or immunosuppressives (52,53). Finally, liver transplantation may be the theoretical best approach in end-stage liver disease, although the evidence is scarce and the survival seems to be decreased in patients with CVID compared with other transplanted patients (54,55).

Our study has some limitations. First, it is a retrospective study with the intrinsic risk of bias of this type of study. Missing data, as well as loss to follow-up patients, and management heterogeneity over time might have consequently occurred. Second, we have excluded patients younger than 18 years, and our results cannot be extrapolated to young patients; however, it should be noted that only 20% of patients with CVID present symptoms younger than 20 years (21). Third, our institution is a reference center for the management of primary immunodeficiencies, which may introduce a selection bias, enriching our series with more severe cases. Therefore, the reported epidemiology of CVID-E and CVID-L and their severity may not be fully representative of the entire spectrum of the disease.

In conclusion, GI and hepatic involvements are common in patients with CVID and frequently occur together. These manifestations significantly affect the disease course, increasing the risk of GI infections, GI malignancy, and, in the case of liver disease, mortality. Patients with both CVID-E and CVID-L disease are believed to be a subgroup of worse prognosis. Prospective multicenter studies are needed to better define these complications and optimize therapeutic strategies.

## CONFLICTS OF INTEREST

**Guarantor of the article:** José Miranda-Bautista, MD, PhD.

**Specific author contributions:** J.M.-B.: designed the study, identified the patients, reviewed the clinical charts, performed the statistical analysis and wrote the manuscript. H.M.-L.: performed the statistical analysis and reviewed the paper. M.D.N.: identified the patients and reviewed the clinical charts. M.A.M.G.: identified the patients, reviewed the clinical charts and reviewed the paper. I.M.-J., P.S.-M. and D.R.: reviewed the paper. M.I.P.G.: reviewed the pathology specimens and reviewed the manuscript. R.B.: designed the study, performed the statistical analysis and reviewed the paper. L.M.: designed the study and reviewed the paper. All authors approved the final version of the manuscript.

**Financial support:** None to report.

**Potential competing interests:** None to report.

**IRB approval statement:** This study does not need an IRB approval.

**Study Highlights**

**WHAT IS KNOWN**

✓ Enteropathy and liver disease are noninfectious manifestations that may occur in common variable immunodeficiency (CVID) patients. Their incidence and prognostic significance remain inadequately characterized.

**WHAT IS NEW HERE**

✓ Intestinal and liver diseases are prevalent in patients with CVID and frequently coexist.
✓ Enteropathy may be the initial clinical presentation of an undiagnosed CVID, acting as a risk factor for subsequent gastrointestinal infections, although it does not contribute to increased mortality risk.
✓ Duodenal atrophy frequently characterizes enteropathy in CVID, correlating with adverse clinical outcomes.
✓ Liver disease is an independent risk factor for increased mortality.

## ABBREVIATIONS:

CVID: common variable immunodeficiency
CVID-E: common variable immunodeficiency-associated enteropathy
CVID-L: common variable immunodeficiency-associated liver involvement
GI: gastrointestinal
HR: hazard ratio
HVPG: hepatic venous pressure gradient
IBD: inflammatory bowel disease
Ig: immunoglobulin
NRH: nodular regenerative hyperplasia
PSVD: porto-sinusoidal vascular disease
